# Development and In Vitro Validation of Antibacterial Paints Containing Chloroxylenol and Terpineol

**DOI:** 10.3390/toxics10070343

**Published:** 2022-06-21

**Authors:** Micaela Machado Querido, Ivo Paulo, Sriram Hariharakrishnan, Daniel Rocha, Nuno Barbosa, Diogo Gonçalves, Rui Galhano dos Santos, João Moura Bordado, João Paulo Teixeira, Cristiana Costa Pereira

**Affiliations:** 1 Environmental Health Department, National Institute of Health, 4000-055 Porto, Portugal; micaelaquerido@hotmail.com (M.M.Q.); cristiana.pereira@insa.min-saude.pt (C.C.P.); 2 EPIUnit, Institute of Public Health, University of Porto, 4050-600 Porto, Portugal; 3 Laboratory for Integrative and Translational Research in Population Health (ITR), 4050-600 Porto, Portugal; 4 Instituto Ciências Biomédicas Abel Salazar, University of Porto, 4050-313 Porto, Portugal; 5 CERENA—Centre for Natural Resources and the Environment, Instituto Superior Técnico, 1049-001 Lisboa, Portugal; ivo.p1691@gmail.com (I.P.); sriram.hariharakrishnan@tecnico.ulisboa.pt (S.H.); diogoazevedogoncalves@ist.utl.pt (D.G.); rui.galhano@ist.utl.pt (R.G.d.S.); jcbordado@ist.utl.pt (J.M.B.); 6 Barbot-Indústria de Tintas, S.A., 4410-295 Vila Nova de Gaia, Portugal; danielrocha@barbot.pt (D.R.); nunobarbosa@barbot.pt (N.B.)

**Keywords:** paint, antibacterial, chloroxylenol, terpineol, cytotoxicity

## Abstract

The establishment of self-disinfecting surfaces is an important method to avoid surface contamination. Recently, paints with antimicrobial properties have been developed to be applied on different surfaces, avoiding contamination with pathogens. In this work, self-disinfecting paints containing Chloroxylenol (CLX), Terpineol (TRP), and a mixture of both substances were developed. The goal was to evaluate and validate these paints using international standards for eventual commercialization and application in scenarios where surface contamination represents a problem. The paints were challenged with five different bacteria, Gram-positive and Gram-negative, before and after a scrub resistance test, where the long-term efficacy of the paints was evaluated. The antibacterial activity assessment was performed following ISO 22196 and JIS Z2801. In general, the paints showed very promising results, demonstrating their antibacterial activity, before and after scrub resistance test. The paint incorporating the mixture of CLX and TRP (CLX+TRP) stood out by revealing consistent results of antibacterial activity both before and after the scrub resistance test for most of the tested bacteria. The cytotoxicity of the developed paints was assessed in vitro by performing tests by direct contact with a human skin cell line, HaCaT, and testes on extracts with HaCaT and a pulmonary cell line, A549. The methodologies for cytotoxicity assessment were developed based in ISO 10993. For genotoxicity assessment, alkaline comet assay was conducted on both cell lines. The cytotoxicity assessment revealed promising results with the paints, demonstrating values of cellular viability above 70% and values of lactate dehydrogenase (LDH) leakage below 30%. The genotoxic assessment also revealed acceptable values of primary DNA damage for the developed antibacterial paints. In general, the selected methodologies presented good potential to be applied in the validation of both efficacy and safety of the antimicrobial paints, aiming to be applied in real scenarios.

## 1. Introduction

Antimicrobial paints have appeared as an alternative solution to prevent and reduce rooms’ walls and other surfaces’ contamination with microorganisms. Recently, different formulations of paints with self-disinfecting properties have been developed [1,2,3].

In the present work, two antimicrobial substances, Chloroxylenol (CLX) and Terpineol (TRP), were selected to engineer paints with self-disinfecting properties. The main goal of this study was to develop and validate antimicrobial paints aiming to be commercialized, with the purpose of being applied in areas with high propensity for infection spreading. To do so, the substances Chloroxylenol (CLX) and Terpineol (TRP) were immobilized onto an acrylic water-based commercial paint, both separately and in a mixture containing the two substances, CLX and TRP (CLX+TRP), since these two antimicrobial substances were already successfully applied together in some formulations, namely in antiseptic soaps [4].

In this work, the antimicrobial substances were functionalized forming a urethane bond. This method was chosen since it allows the formation of strong urethane covalent bonds. Covalently bonding the antimicrobial substances to the polymeric matrix restricts the possibility of diffusion, increasing the durability of the antimicrobial properties and decreasing the risk of absorption through contact or environmental release [5,6].

CLX is a chlorinated phenolic compound well known for its application as an antimicrobial substance in hygiene products, cosmetics, and antiseptics, among others. This substance is also used as a disinfectant, being applied in several wound cleaners, household disinfectants, and surgical instrument disinfection solutions [4,7,8,9]. CLX has proven antimicrobial efficacy against several bacteria and fungi [10,11,12]. Regarding the incorporation of CLX on surfaces, in a study by Mansouri et al., urinary catheters were impregnated with CLX and tested in vitro against several urinary pathogens. The antimicrobial catheters showed a broad-spectrum activity against different bacteria and fungi. Moreover, these antimicrobial catheters were also tested in vivo using rabbit models and showed very interesting results with the CLX-impregnated catheters revealing a contamination rate of only 12.5%, while the controls (regular catheters) showed rates of contamination of 62.5% [10].

TRP is a monocyclic monoterpene naturally present in several plants, such as flowers and pines. TRP has five different isomers, alfa (α), beta, gamma, delta, and terpinene-4-ol. α-TRP, the one we used in this work, is one of the most common isomers found in nature, and it is frequently applied in several products such as cosmetics, perfumes, and in pharmaceutical products. More recently, medical properties have also been associated with TRP, namely antioxidant activity, anticancer properties, anticonvulsant activity, cardiovascular effects, and antimicrobial properties [13,14]. TRP has proved antibacterial and antifungal properties against several microorganisms [15,16,17]. 

The antibacterial activity of antimicrobial paints is frequently evaluated according to the international standards ISO 22196 and JIS Z2801, which evaluate the antibacterial activity of polymeric and non-porous materials [18,19]. In this work, those international standards were followed, and five different bacteria were tested. *Staphylococcus aureus* (*S. aureus*) and *Escherichia coli* (*E. coli*) were selected due to being considered standard bacteria and, thus, recommended by ISO 22196 and JIS Z2801 and for being frequently associated with surface contamination on healthcare facilities [20]. In addition, three more species, frequently associated with hospital-acquired infections or environmental contamination, were tested: *Bacillus cereus* (*B. cereus*), *Enterococcus faecalis* (*E. faecalis*), and *Klebsiella variicola* (*K. variicola*) [21,22].

The toxicity assessment of the paints towards human models is a crucial step to assure their safety; nevertheless, this important procedure is frequently absent in research studies reporting the development of antimicrobial paints.

In this study, the toxicity evaluation approach was based on ISO 10993:5, which establishes the methodology for the in vitro assessment of the cytotoxicity of medical devices and other materials [23], being also frequently used to assess the toxicity of antimicrobial coatings and materials [24,25]. Following the recommendations of ISO 10993:5, tests by direct contacts and tests on extracts were performed for cytotoxicity evaluation. After the tests, a quantitative evaluation of the cellular viability and cell lysis was performed.

The genotoxicity potential of the paints was also tested by performing the comet assay after exposing, in vitro, human cell lines to the paints. In the cyto- and genotoxicity assessment, human skin cells, more specifically keratinocytes (HaCaT cell line) and human alveolar pulmonary cells (A549 cell line) were used. These cell lines were selected accordingly to two of the main routes of environmental exposure to the paints, inhalation and dermal absorption [26,27]. 

## 2. Materials and Methods

### 2.1. Chemicals

Chloroxylenol (CLX) (CAS No. 88-04-0) was purchased from Acros Organics (Geel, Belgium). Tryptic soy agar (TSA), maximum recovery diluent (MRD), tryptone soya broth (TSB), and plate count agar (PCA) were purchased from VWR, (Radnor, PA, USA). Dulbecco’s modified Eagle’s medium (DMEM) with 4.5 g/L glucose and 2 mM L-glutamine and Trypsin-ethylenediaminetetraacetic acid (Trypsin-EDTA) 0.25%/1 mM EDTA2Na in Hank’s balanced salt solution (HBSS), w/o:Ca and Mg, w: Phenol red were acquired from PanBiotech (Aidenbach, Germany). Antibiotic-antimycotic (100×) solution and phosphate-buffered saline (PBS) 10× Molecular Biology Grade were purchased from Corning (Corning, NY, USA). Fetal bovine serum heat inactivated (FBS) was bought from Biowest (Nuaille, France). Triton X-100 (CAS No. 9002-93-1), low melting point (LMP) agarose (CAS No. 39346-81-1), ethylenediaminetetraacetic acid disodium salt dihydrate (Na_2_EDTA) (CAS No. 6381-92-6), dodecylbenzenesulfonic acid sodium salt (CAS No. 25155-30-0), Neutral Red (CAS No. 553-24-2), and acridine orange (CAS No. 494-38-2) were purchased from Sigma-Aldrich (St. Louis, MI, USA). The water-soluble tetrazolium (WST-1) cell proliferation reagent kit (CAS No.150849-52-8) and lactate dehydrogenase (LDH) cytotoxicity detection kit were purchased from Roche (Basel, Switzerland). Sodium chloride (NaCl) (CAS No. 7647-14-5) and InvitrogenTM SYBR^®^ Gold solution were bought from Thermo Fisher Scientific (Waltham, MA, USA). Normal melting point (NMP) agarose was supplied by Bioline (London, UK). Dimethyl sulfoxide (DMSO) (CAS No. 67-68-5) was purchased from Honeywell (Seelze, Germany). Tris hydrochloride (Tris HCl) (CAS No.1185-53-1), tris base (CAS No. 77-86-1), sodium hydroxide (NaOH) (CAS No. 1310-73-2), methyl methanesulfonate (MMS) (CAS No. 66-27-3), and sodium lauryl sulfate (SLS) (CAS No. 151-21-3) were bought from Merck KGaA (Darmstadt, Germany). 

### 2.2. Paint Preparation

As previously noted, the substances CLX and TRP were purchased. The paints were prepared according to the procedure disclosed in Silva et al. [5] and Querido et al. [28].

Briefly, after the derivatization, the substances CLX and TRP were added to a commercial water-based acrylic paint. The mixing was performed in a mechanical stirrer with shear force at 800 rpm speed for 5 min at RT and humidity conditions (25 °C, 50% HR).

The CLX and TRP were mixed at 0.15 g/L and 6.0 g/L, respectively; the combination of the two substances, CLX+TRP, was mixed at 3.0 g/L. During the optimization process of paint preparations, different criteria were taken into account—namely, the paints’ color, viscosity, and opacity. 

After incorporation of the different substances, the paint color remained similar to the original. After application on a clear-coated opacity chart (2A-H from Leneta) (Mahwah, NJ, USA) and drying during 24 h at room temperature, no differences were found in the color compared with the original paint. The values of whiteness also were similar compared to the standard paint without the substances. The physical properties of the paints only suffered minor modifications after incorporation of the antimicrobial substances. The viscosity of the final formulations, containing CLX, TRP, and CLX+TRP were slightly higher (355 cP, 358 and 364 cP, respectively) compared with the original paint (312 cP). The values of opacity were also similar for the three formulations, with the original paint presenting a value of 99.28%, CLX a value of 99.18%, TRP a value of 99.11%, and CLX+TRP a value of 99.25%. 

The equipment used for measuring the viscosity was Stormer Viscometer Myr VK2000 by Viscotech Hispania S.L. (Tarragona, Spain), and opacity and whiteness were analyzed using a DC400 spectrophotometer by Datacolor (Lawrenceville, NJ, USA). For utilization of our assays, the final formulations were then applied on polymeric coupons (50 mm × 50 mm or 10 mm × 10 mm), forming a layer of 200 µm of thickness. The drying time of the paint after application was 24 h.

### 2.3. Functionalised Antimicrobial Molecules

Fourier Transform Infrared Spectroscopy (FTIR) analysis in PerkinElmer FTIR Spectrometer coupled to an attenuated total reflectance (ATR) unit from PerkinElmer with an individual Diamond crystal was performed with both antimicrobial substances and their functional counterparts. Studies were carried out in a frequency range of 600–4000 cm^−1^ with 4 cm^−1^ resolution. FTIR-ATR also evaluated the reaction progress.

### 2.4. Samples Preparation

The samples used in the tests were the unmodified acrylic water-based paint (Un_Paint) and the self-disinfecting paints containing CLX in the concentration 0.15 g/L, TRP in the concentration of 6.0 g/L, and the mixture CLX+TRP in the concentration of 3.0 g/L. For antibacterial assessment, the paints were applied in 50 × 50 mm polymeric film square, according to ISO 22196 [18]. For cytotoxicity assessment, the paints were applied in a 10 × 10 mm polymeric film square. Before each test, every sample and parafilm were sterilized with UV-C light (294 nm) using a UV lamp from VWR (Radnor, PA, USA) for 15 min on each side.

For the tests by direct contact, samples of transparent polymeric film (W) and Copper (Cu^2+^) were used as negative and positive control of the surface, respectively. 

### 2.5. Antibacterial Activity

*E. coli* (ATCC 25922), *K. variicola* (ATCC 31488), *S. aureus* (ATCC 25923), *B. cereus* (isolated and identified from samples processed in our laboratory), and *E. faecalis* (NCTC 775) were issued in antibacterial assessment. 

Antibacterial activity of the paints was assessed following ISO 22196 and JIS Z2801, with minor modifications [18,19], as described by Querido et al. [29]. The bacteria were grown as previously described and the inoculum was prepared with the concentration of 6 × 10^5^ colony forming units per milliliter (CFUs/mL).

Briefly, the samples of Un_Paint, CLX, TRP or CLX+TRP were placed on sterile petri dishes, inoculated with 400 μL of bacterial inoculum, and covered with parafilm (40 × 40 mm) followed by 24 h incubation at 37 °C, with high humidity levels. Afterwards, 10 mL of a TSB-neutralizing solution was added to each petri dish, the parafilm was removed, and several dilutions, from 10^−1^ to 10^−5^, were made using MRD. After, 15 mL of previously melted PCA was added to each petri dish. After drying, the plates were incubated for 48 h at 37 °C followed by the counting of the number of CFUs on each plate and the calculation of the number of viable bacteria per cm^2^.

Following Equation (1), described in ISO 22196 and JIS Z 2801 [18,19], the value of antibacterial activity (R) was obtained and according to the values found for R, the samples were classified as having antibacterial activity (R ≥ 2) or not (R < 2):

(1) $$R=\left(U_{t}-U_{0}\right)-\left(A_{t}-U_{0}\right)=U_{t}-A_{t}$$

where *R* is the antibacterial activity; *U*_0_ is the average of the common logarithm of the number of viable bacteria, in CFUs/cm^2^, recovered from the control paint samples immediately after inoculation (T0); *U_t_* is the average of the common logarithm of the number of viable bacteria, in CFUs/cm^2^, recovered from the control paint samples after 24 h (T24); A_t_ is the average of the common logarithm of the number of viable bacteria, in CFUs/cm^2^, recovered from the antibacterial paint samples after 24 h (T24).

### 2.6. Scrub Resistance Test

The wet scrub resistance was evaluated according with the ISO 11998 [30]. The three different paints were applied in PVC foils from Leneta (Mahwah, NJ, USA) (with a wet thickness of 400 μm. After 28 days of drying at room temperature, a solution of dodecylbenzenesulfonic acid sodium salt in water (2.5 g/L) was used to wet the surfaces containing the paints. Afterwards, the scrub resistance test was performed on the Abrasion and Washability Tester Model 1720M004 from Elcometer (Manchester, UK) using a scrub pad (“3 M Scotch Brite”, No. 7448, Type S, Grade UFN, Grey). After 200 cycles of washing, the Leneta foils with the paints were rinsed with tap water to remove any residues that could be presented in the surface. 

After scrub resistance test, the antibacterial activity was assessed as described in Section 2.5.

### 2.7. Cytotoxicity

According to ISO 10993, tests by direct contact and tests on extracts may be used to assess the in vitro cytotoxicity of medical devices or other materials for close contact with the users [23]. 

In the tests by direct contact, a portion of the material to be tested is placed in close contact with the cellular models. In the tests on extracts, the tested material is lixiviated using an extraction vehicle to obtain extracts. These pure extracts, as well as several dilutions are used to determine their in vitro effect in the cell line models and assess a potential a dose–response effect. In both tests, after exposure to the cells and incubation for at least 24 h, a quantitative evaluation of cytotoxicity was performed by measuring parameters such as cellular viability. According to ISO 10993:5, a reduction of cellular viability by more than 30% is considered a cytotoxic effect. Different assays are suggested by the ISO 10993:5 to measure the cytotoxicity, namely the neutral red uptake (NRU) cytotoxicity assay or the 3-(4,5-Dimethylthiazol-2-yl)-2,5-Diphenyltetrazolium Bromide (MTT) assay [23]. In this work we performed NRU assay as suggested, WST-1 cell proliferation reagent (WST-1) assay, an analogous test of MTT and Lactate Dehydrogenase (LDH) release assay, which evaluates cells’ membrane integrity [31].

#### 2.7.1. Cell Culture

HaCaT cells, a nontumorigenic immortalized human keratinocyte cell line, was obtained from Cell Lines Service (Eppelheim, Germany). The A549 cell line, a human alveolar epithelial cell line (ECACC 86012804; Human Caucasian lung carcinoma) was purchased from the European Collection of Authenticated Cell Cultures (ECACC, Salisbury, UK). 

Cells were cultured in complete medium (DMEM supplemented with 10% (*v*/*v*) FBS and 1% (*v*/*v*) antibiotic-antimycotic solution) and grown at 37 °C, 5% CO_2_ in humidified atmosphere. The medium was changed every two days and culture was split when 80% confluency was reached using 0.25% trypsin-EDTA to detach cells. 

#### 2.7.2. Tests by Direct Contact

Tests by direct contact were performed as previously described [29] in a HaCaT cell line model.

Briefly, HaCaT cells were seeded at a concentration of 1.0 × 10^5^ cells/mL in 6-well plates (2 mL) and adhered for 24 h at 37 °C, 5% CO_2_ in a humidified atmosphere.

After cell incubation, culture medium was replaced with fresh assay medium (DMEM + 5% FBS) and the samples (Un_Paint, CLX, TRP, CLX+TRP, W, and Cu^2+^) were placed over the cell layer and gently pushed to contact directly with the cells. The 6-well plates were then incubated for 24 h at 37 °C, 5% CO_2_ in a humidified atmosphere. 

Afterwards, the samples were gently removed from the surface of the cells and cellular viability and membrane integrity were assessed using WST-1, NRU, and LDH assays, performed as previously described [29]. Assay medium was used as negative control for the three assays. Triton X-100 solution (1%) was used as positive control for WST-1 and LDH and sodium lauryl sulfate (SLS) 0.2 mg/mL was used as positive control for the NRU assay.

Microscopic observations were performed using an IT 400 inverted microscope by VWR (Radnor, PA, USA) in order to verify the cellular growth and morphology after 24 h of contact with the paint samples.

The exposures were performed in triplicates on three independent experiments.

#### 2.7.3. Tests on Extracts

Tests on Extracts, were performed as previously described [29], in HaCaT and A549 cells.

Briefly, the samples were placed in 24-well plates and 1 mL of assay medium (DMEM + 5% FBS) was added, followed by 24 h of incubation at 37 °C, 5% CO_2_, to allow the leaching of the chemicals from the samples. Simultaneously, cells were seeded at a concentration of 1.0 × 10^5^ cells/mL in 96-well plates (100 μL) and incubated for 24 h for adhesion at 37 °C, 5% CO_2_ in a humidified atmosphere.

After 24 h, different dilutions of the released extracts were performed. The original extracts (100%) were diluted to 75%, 50%, and 25%. Then, the cells’ medium was replaced with freshly prepared extracts (100%, 75%, 50%, and 25%). The cells were exposed for 24 h, at 37 °C, 5% CO_2_ in a humidified atmosphere, and afterwards, cellular viability and membrane integrity were assessed by WST-1, NRU, and LDH assays, performed as previously described [29]. Assay medium was used as negative control for the three assays. Triton X-100 solution (1%) was used as positive control for WST-1 and LDH and sodium lauryl sulfate (SLS) 0.2 mg/mL was used as positive control for NRU assay.

The exposures were performed in triplicates on three independent experiments.

### 2.8. Genotoxicity

#### Alkaline Comet Assay

Before performing the comet assay, the cells were exposed according to the protocol by Querido et al. [29]. Briefly, the cells, HaCaT and A549, were grown for 24 h in 24-well plates and afterwards the medium was replaced by new complete medium containing different concentrations of extracts (100% and 25%). Then, the plates were incubated for 24 h at 37 °C, 5% CO_2_. Complete medium was used as negative control and a solution of MMS (800 µM) was used as a positive control. Three replicates of each condition were prepared. The cells were frozen as previously described and kept at −80 °C until comet assay was performed.

The alkaline comet assay was then performed following a medium throughput 12-gel comet assay protocol, according to Querido et al. [29].

Briefly, after gels and slides preparations as previously described, the slides were washed with PBS for 5 min and immersed in electrophoresis solution (Na_2_EDTA 1 mM, NaOH 0.3 M, pH 13) for 30 min at 4 °C followed by electrophoresis that was performed for 30 min at 18 V. Afterwards, the slides were washed and fixed, being left to dry overnight. For microscopic evaluation, the slides were stained with 1:10,000 dilution of SYBR^®^ Gold in TE buffer (Tris−HCl 10 mM and EDTA 1 mM, pH 7.5–8) and observed using a Motic BA410 ELITE Series microscope, equipped with an EPI-fluorescence kit, with 100× magnification and the comets were scored using the software Comet Assay IV image analysis software (Perceptive Instruments, Staffordshire, UK). At least 100 cells in each sample (50 cells/nucleoids in each gel) were scored.

### 2.9. Statistical Analysis

At least three replicates were used in each independent experiment that was repeated three times. The data from the three independent experiments were analyzed together. Data are reported as mean ± standard deviation (SD).

For antibacterial activity assessment, before and after scrub resistance tests, the statistical differences of data against negative control (Un_Paint) were analyzed by two-way ANOVA followed by Sidak’s multiple comparison test.

In the tests on extracts, tests by direct contact, and alkaline comet assay, statistical significances of data against negative control were analyzed by one-way ANOVA followed by a Dunnett post hoc test.

In tests by direct contact, statistical differences between different paints were analyzed by one-way ANOVA followed by Tukey’s test.

In alkaline comet assay, the differences between different paints in the same extract concentration were analyzed by two-way ANOVA followed by Tukey’s test.

Statistical differences between the same concentration of the same paint, but for different cell lines (comparing A549 and HaCaT), were analyzed by two-way ANOVA followed by Sidak’s multiple comparison test.

Data were tested for normality and homogeneity of variances by Shapiro–Wilk and Bartlett’s tests, respectively.

The differences were considered statistically significant for *p* < 0.05. The statistical analyses were performed using Graph Pad Prism version 8.0 (GraphPad Software, San Diego, CA, USA, 2018).

## 3. Results

### 3.1. Isocyanate Functional Antimicrobial Substances

**Figure 1:**
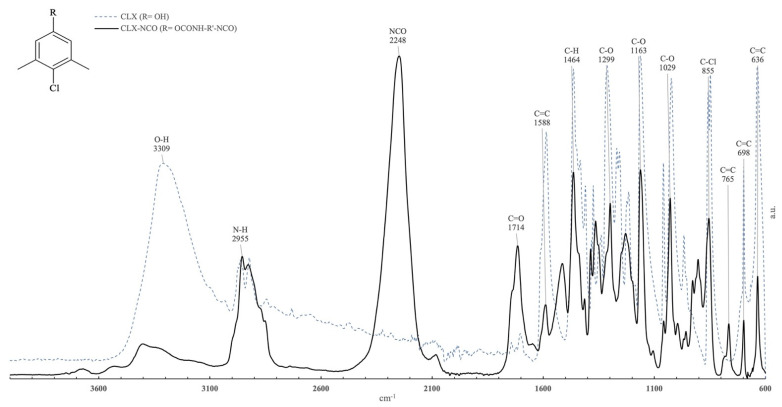
Normalized absorbance infrared spectra (FTIR-ATR) obtained from Chloroxylenol (dashed-line blue, R = OH), and its isocyanate functional derivative (solid line, R = OCONH–R′–NCO, C–NCO).

**Figure 2:**
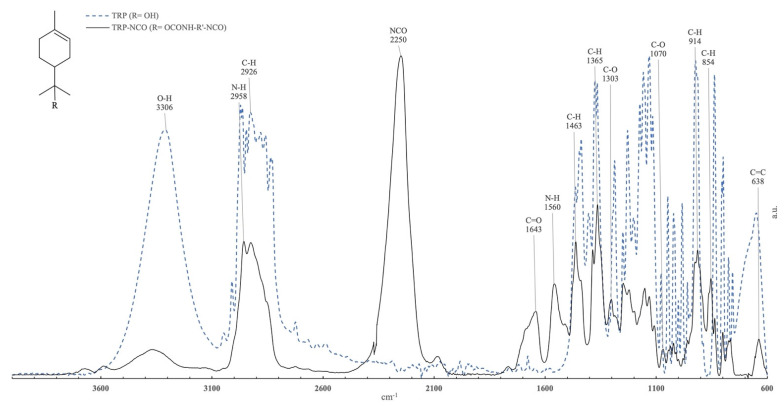
Normalized absorbance infrared spectra (FTIR-ATR) obtained from a-Terpineol (dashed-line, R = OH), and its isocyanate functional derivative (solid line, R = OCONH–R′–NCO, T–NCO).

The commercial antibacterial substances were functionalized with isophorone diisocyanate (IPDI). Conversions as high as 95% ± 5% were obtained. Immobilization occurred by forming a urethane group between the OH group, of both CLX and TRP, with one of the NCO groups of IPDI. The success of the urethane formation was confirmed by FTIR-ATR, as shown in Figure 1 and Figure 2.

In Figure 1 it is possible to observe the main characteristic groups of CLX and the isocyanate functionalized CLX. The observed spectrum range between 3590 cm^−1^ and 3095 cm^−1^ was assigned to the OH group. While, the peaks in the range between 1588 cm^−1^ and 636 cm^−1^ were associated with the C–H, C–O, and C=C bonding from the CLX ring structure. The peak at 1464 cm^−3^ was associated with the C–H bonding of the substituent methyl groups. At 855 cm^−1^ the C–Cl bonding was observable. 

With the functionalization of CLX, new bands appeared, such as the characteristic band located at 2248 cm^−1^ and assigned to the N=C=O isocyanate stretch, which confirmed the functionalization effectiveness. The functionalization success is also visible by the appearance of the assigned C=O stretch band located at 1714 cm^−1^, corroborating bond formation between the isocyanate function and the OH group from CLX. The reduction of the intensity of the OH group band and the change of appearance of a peak at 2955 cm^−1^ due to the formation of a secondary amine is also observable.

In Figure 2 it is possible to observe the main characteristic groups of TRP and the isocyanate functionalized TRP. The observed spectrum range between 3524 cm^−1^ and 3105 cm^−1^ was assigned to the OH group. The peaks in the range between 1463 cm^−1^ and 854 cm^−1^ were associated with the C–H and C–O groups from the TRP structure. At 638 cm^−1^ it was possible to identify the peak representing the C=C bonding.

For the functional TRP, similar to the CLX new bands, the characteristic band was located at 2250 cm^−1^ and assigned to the N=C=O isocyanate stretch; the C=O stretch band was at 1643 cm^−1^, and again the change of appearance of a peak at 2958 cm^−1^ was observable due to the formation of a secondary amine, confirming the functionalization effectiveness.

### 3.2. Antibacterial Activity 

**Figure 3:**
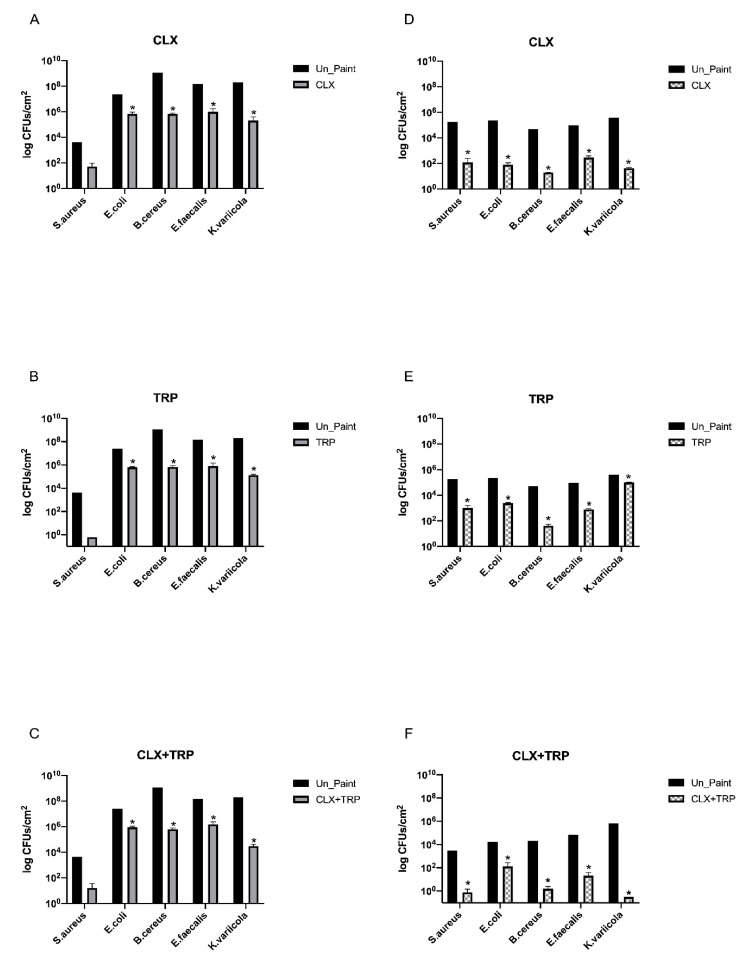
Average number of viable CFUs/cm^2^ recovered from each antimicrobial paint before (**A**–**C**) (left) and after (**D**–**F**) (right) scrub resistance test followed by 24 h of incubation. The values are presented in logarithmic scale. The values are expressed as mean ± standard deviation. The statistical significance of samples (CLX/TRP/CLX+TRP) compared with Un_Paint is represented by *. (Two-way ANOVA; *p* < 0.05).

The results of antibacterial activity assessment before scrub resistance test showed that the three paints were able to significantly reduce the number of CFUs/cm^2^ after incubation with bacteria for 24 h (Figure 3A–C). 

According to the ISO 22196 criteria [18], before scrub resistance tests, the paint CLX presented antibacterial activity (R ≥ 2) against all tested bacteria (Table 1). TRP presented antibacterial activity against *S. aureus*, *E. coli*, *B. cereus*, and *E. faecalis* but not against *K. variicola.* CLX+TRP also demonstrated antibacterial activity against all tested bacteria. 

After scrub resistance test, reductions on CFUs/cm^2^ were observed for all the tested paints and with all tested bacteria (Figure 3D–F). The three paints, CLX, TRP, and CLX+TRP, demonstrated to have antibacterial activity (R ≥ 2) against *S. aureus*, *B. cereus*, *E. faecalis*, and *K. variicola*. Only for *E. coli* did the values of antibacterial activity not fulfill the criteria of being equal or superior to 2, even if a reduction in the number of CFUs/cm^2^ was verified.

### 3.3. Cytotoxicity

As previously explained, the cytotoxicity evaluation followed two different methodologies, namely the tests by direct contact and tests on extracts.

**Figure 4:**
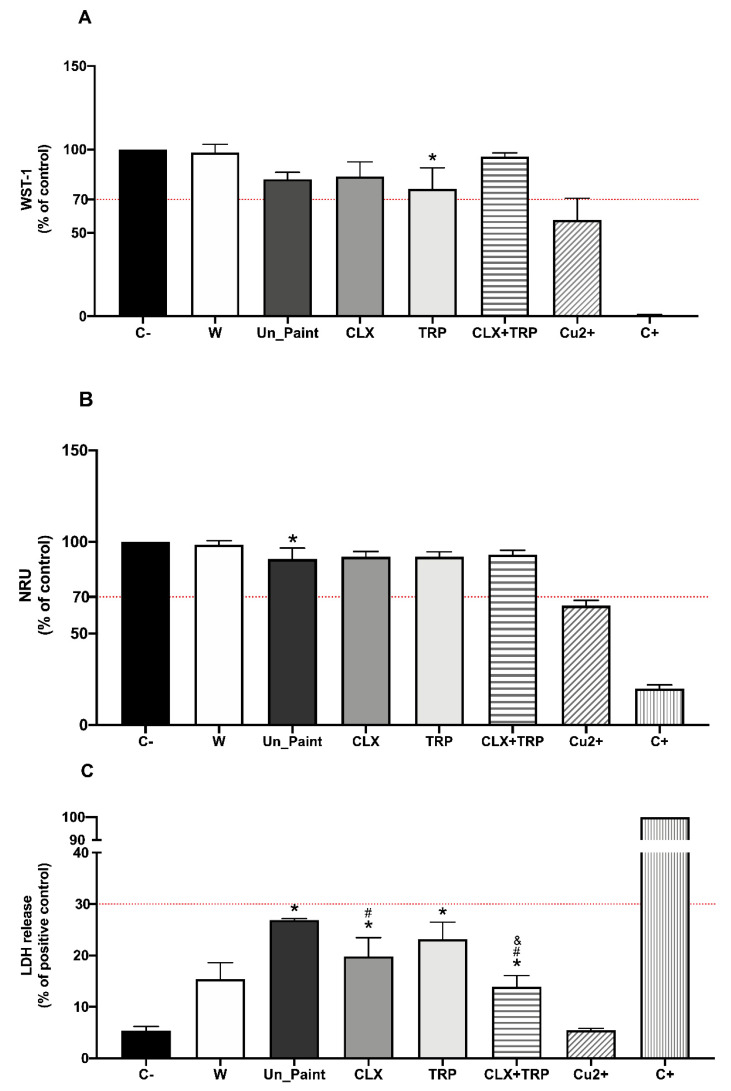
Results of WST-1 (**A**), NRU (**B**), and LDH (**C**) assays, with HaCaT cells, after 24 h of direct contact with Un_Paint, CLX, TRP, CLX+TRP, transparent polymeric film (W), and Copper (Cu^2+^). C− negative control (assay medium), C+ positive control (Triton X-100 solution (1%) for WST-1 and LDH, or SLS 0.2 mg/mL of NRU). The red lines represent the defined thresholds of acceptable values for each parameter, 70% for cellular viability and 30% for LDH leakage. The values are expressed as mean ± standard deviation. The statistical significance of samples compared to C− is represented by *, the statistical differences compared to Un_Paint are represented by #, and the statistical differences compared to TRP are represented by &. (One-way ANOVA; *p* < 0.05).

In the tests by direct contact (Figure 4), CLX presented higher values of cellular viability than Un_Paint, both with WST-1 assay (83.86 ± 8.72% for CLX vs. 82.19 ± 4.13% for Un_Paint) and with NRU assay (91.86 ± 2.83% for CLX vs. 90.52 ± 6.06% for Un_Paint). TRP was the only paint that presented a significant decrease in cellular viability (76.32% ± 12.64%) comparing to the negative control in WST-1 assay; however, with NRU assay this paint presented higher values of viability 91.82 ± 2.73%.

The paint with higher values of cellular viability was CLX+TRP, showing a viability of 95.72 ± 2.19% with WST-1 and of 92.98 ± 2.47% with NRU.

Concerning the LDH release assay, all the paints presented significant increases in LDH leakage comparing with the negative control; however, none of the paints reached the established limit of 30%. In CLX, a release of 19.80 ± 3.66% was observed, with this paint being statistically different from the control and from the Un_Paint (26.89 ± 0.31%). TRP had a release of 23.19 ± 3.27% and CLX+TRP of 13.93 ± 2.13%. CLX+TRP was the paint with lower values of LDH release, revealing statistic differences from the negative control, from the Un_Paint, and from TRP.

Regarding the microscopic observation after incubation of the paints in direct contact with the HaCaT cells, alterations in cellular growth and morphology were not detected. Comparing with the negative control, a reduction in cellular density was observed in the presence of the paints (images in the Supplementary Materials, Figure S1).

**Figure 5:**
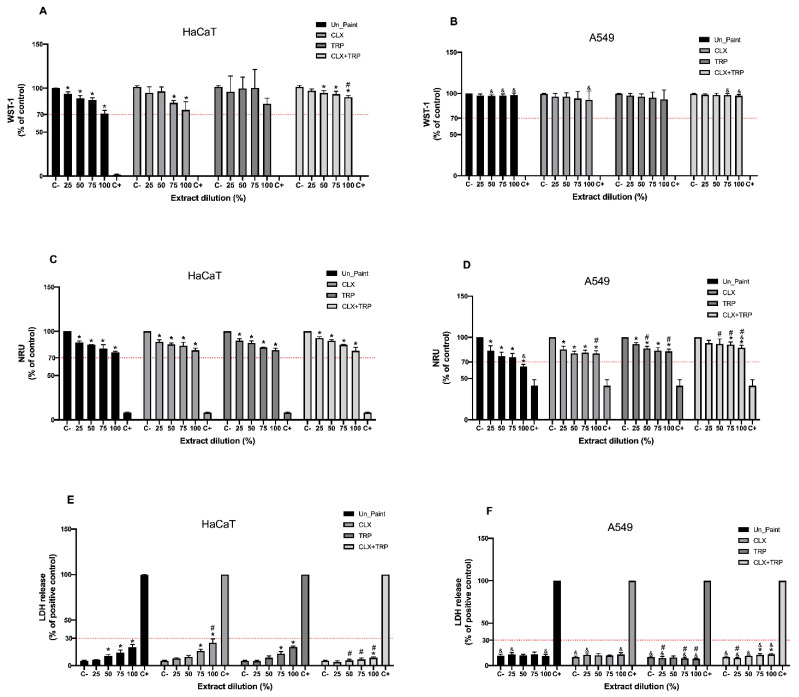
Results of WST-1 (**A**,**B**), NRU (**C**,**D**), and LDH (**E**,**F**) assays, with HaCaT and A549 cells, after 24 h incubation with Un_Paint, CLX, TRP, and CLX+TRP extracts at concentration of 25%, 50%, 75%, and 100%. C− negative control (assay medium), C+ positive control (Triton X-100 solution (1%) for WST-1 and LDH, or SLS 0.2 mg/mL solution of NRU). The red lines represent the defined thresholds of acceptable values for each parameter, 70% for cellular viability and 30% for LDH leakage. The values are expressed as mean ± standard deviation. The statistical significance of the extracts compared to C− is represented by * (One-way ANOVA; *p* < 0.05) and the statistical differences compared to Un_Paint are represented by # (Two-way ANOVA; *p* < 0.05). The statistical significance of the extract on A549 cells compared to the same extract, in the same concentration, on HaCaT cells is represented by & (Two-way ANOVA; *p* < 0.05).

In the tests on extracts (Figure 5), the WST-1 assay with HaCaT cells revealed that after exposure to CLX paint, the cells displayed a decrease in cellular viability, reaching statistical difference from the control for 75% (83.28 ± 2.47%) and 100% (75.22 ± 9.31%) extracts. The TRP did not present statistical differences from the control and CLX+TRP presented statistical differences for the 50% (94.55 ± 2.76%), 75% (93.17 ± 3.35%), and 100% (89.82 ± 1.89%) extracts.

In the NRU assay, the CLX, TRP, and CLX+TRP paints presented dose-dependent effects and disclosed statistical differences from the control for all concentrations. 

The membrane integrity assay with HaCaT cells also revealed a dose-dependent response for all the paints. The CLX paint showed a significant increase in LDH release for the concentrations 75% (16.09 ± 1.97%) and 100% (25.18 ± 4.15%). The same tendency was verified for the TRP, which also presented statistical differences for the 75% (13.19 ± 2.31%) and 100% (20.57 ± 1.03%) extracts. The CLX+TRP displayed lower values of LDH release, with 100% (8.69 ± 0.95%) extract being the only concentration presenting statistical difference from the negative control.

Regarding the WST-1 assay performed with A549 cell line, no significant differences were found between the paints and the negative control. In the NRU assay, however, significant differences in all the paints were detected. 

The CLX paint presented significant differences from the negative control in all extracts’ concentration, as well as TRP. However, for CLX+TRP only 75% (91.07 ± 3.31%) and 100% (87.09 ± 3.07%) extracts presented significant reductions on cellular viability. 

In the LDH assay with A549 cells, only CLX+TRP presented values statistically different from the negative control for 75% (12.67 ± 1.43%) and 100% (13.17 ± 1.02%) extracts.

### 3.4. Genotoxicity

Following the exposure of extracts (25% and 100%) to the cells, primary DNA damage was evaluated, in HaCaT and A549 cell lines, by alkaline comet assay. The chosen descriptor was % tail intensity that measures the % of DNA in the tail [32]. 

**Figure 6:**
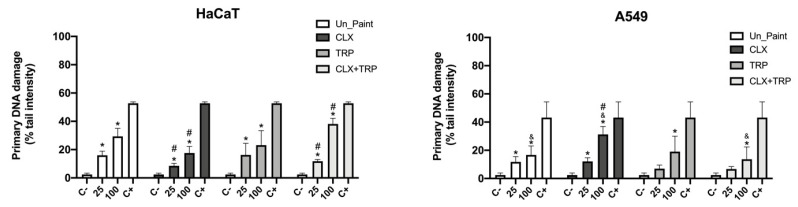
Results of alkaline comet assay, with HaCaT (**left**) and A549 (**right**) cell lines, after exposure to Un_Paint, CLX, TRP, or CLX+TRP extracts for 24 h. C− negative control (complete medium), C+ positive control (MMS 800 μM solution). The values are expressed as mean ± standard deviation. The statistical significance of samples compared to C− is represented by * (One-way ANOVA; *p* < 0.05). The statistical significance of samples compared to Un_Paint is represented by # (Two-way ANOVA; *p* < 0.05). The statistical significance of the extract on A549 cells compared to the same extract, in the same concentration, on HaCaT cells is represented by & (Two-way ANOVA; *p* < 0.05).

Concerning the primary DNA damage, a very pronounced dose-dependent effect was verified for the HaCaT cell line after exposure to the paints (Figure 6). All the paints presented a significant increase in single-strand DNA breaks compared with the negative control. CLX presented lower values (8.56 ± 1.81% for CLX 25% and 17.60 ± 4.67% for CLX100%) of single-strand DNA breaks compared with the Un_Paint (16.02 ± 2.90% for Un_Paint25% and 29.35 ± 5.67% for Un_Paint100%). 

TRP presented values similar (16.25 ± 8.18%) to Un_Paint 25%; however, it presented lower values (23.17 ± 10.26%) of primary DNA damage in the original extract at 100%. On its turn, CLX+TRP revealed a decrease in single-strand DNA breaks for the 25% extract (11.80 ± 1.20%) relative to the Un_Paint; however, for the 100% extract it was the paint with higher values of primary DNA damage (38.16 ± 3.99%).

After exposure in A549 cell line, there were increases in percentage of tail intensity for all the paints compared with the negative control, however with statistical significance only for CLX25%, CLX100%, TRP100%, and CLX+TRP100%. CLX100% and TRP100% presented higher values (31.27 ± 5.59% for CLX100% and 19.02 ± 11.00% for TRP100%) of single-strand DNA damage than the Un_Paint100% (16.73 ± 6.30%). However, CLX+TRP revealed a decrease in primary DNA damage for both extracts’ concentration, 25% (6.71 ± 1.75%) and 100% (13.64 ± 8.76%).

Comparing the two cell lines, A549 presented statistically significant differences from HaCaT for Un_Paint100%, CLX100%, and CLX+TRP100%.

## 4. Discussion

The development of paints with antimicrobial properties has shown promising outcomes. Different paint formulations have been recently developed, either containing natural or synthetic materials or substances with antimicrobial properties [1,2,3]. 

In our study, through the analysis of the FTIR spectrum, it was possible to observe the formation of a urethane bonding formed by the OH group of CLX and TRP and the NCO group if IPDI, attesting to the successful functionalization. Additionally, the NCO band still visible on the CLX-NCO and TRP-NCO indicates that there were still NCO groups available for functionalization with the paint matrix. The FTIR analysis also allowed us to verify the successful functionalization of the substances CLX and TRP, and the successful immobilization of these substances on the acrylic paint. The presence of characteristic peaks of both substances was verified in FTIR spectrum.

Regarding our results obtained for antibacterial activity before scrub resistance test, most of the formulated paints displayed antibacterial activity, significantly reducing the number of viable bacteria after 24 h. CLX reduced the number of CFUs/cm^2^ by more than 3 log for *S. aureus*, *E. coli*, and *B. cereus.* For *K. variicola*, the reduction was even higher, around 4 log. This paint presented values of R ≥ 2, meaning that the paint has antibacterial activity against all the tested bacteria. A recent study from Weldrick et al. already demonstrated the antibacterial properties of CLX against biofilms of *S. aureus* [33]. Another previous study from Perez-Garza et al. demonstrated the antibacterial activity of CLX by eliminating *E. coli* and *E. faecalis* from contaminated hands and reducing the microbial load in water rinsates after wash with antimicrobial soap containing CLX [34]. 

For TRP, a reduction in the number of CFUs/cm^2^ by more than 2 log was observed for *S. aureus*, *E. coli*, and *E. faecalis.* For *B. cereus*, the reduction was of around 3 log; however, for *K. variicola* the reduction was inferior to 1 log. This being, the TRP paint was demonstrated to have antibacterial activity against *S. aureus*, *E. coli*, *B. cereus*, and *E. faecalis*, but it did not fulfill the criteria to be considered antibacterial against *K. variicola.*

A study by Guimarães et al. proved the bactericidal properties of TRP against *S. aureus* and the bacteriostatic effects against *B. cereus* and *E. coli*. In this study, the minimum inhibitory concentration (MIC) of TRP for *S. aureus*, *B. cereus*, and *E. coli* were assessed, revealing values of 0.03 mg/mL, 0.12 mg/mL, and 0.06 mg/mL, respectively. For *S. aureus*, the minimum bactericidal concentration (MBC) found was 0.12 mg/mL [35]. According to Freire et al., the MIC and MBC found for TRP against *E. faecalis* was 2 mg/mL, for both [36]. Moreover, in a very recent study, α-TRP was incorporated in poly(butylene adipate terephthalate) and poly(lactic acid) biodegradable films produced for food packaging. The results showed that the films containing TRP presented a reduction on total bacteria count compared with the control (regular films without TRP) after 3, 6, 9, and 12 days of packaging at 4 °C [37]. 

The paint containing the mixture CLX+TRP significantly reduced the number of viable bacteria after 24 h incubation for all the tested bacteria. For *S. aureus*, *E. faecalis*, and *B. cereus* the reduction was by around 4 log, while for *E. coli* it was of around 3 log and for K. variicola the reduction was even more pronounced, around 6 log. 

In general, before scrub resistance test, CLX+TRP was the paint that presented better antibacterial effects, being the paint that presented more consistency of results regardless of the tested bacteria. However, after scrub resistance tests, some modifications were detected on the results. CLX was able to significantly reduce the number of CFUs/cm^2^ for *E. coli*, *B. cereus*, *E. faecalis*, and *K. variicola*; however, for *S. aureus*, the reduction was not statistically significant. Nonetheless, the CLX paint was still considered to have antibacterial activity against *S. aureus*, *B. cereus*, *E. faecalis*, and *K. variicola.* The values of antibacterial activity, R, for CLX before and after scrub resistance test, were similar for *B. cereus* and *E. faecalis*; however, for *S. aureus*, *E. coli*, and *K. variicola*, there was a reduction in antibacterial activity of 1.3 for *S. aureus*, 1.9 for *E. coli*, and 0.9 for *K. variicola*. TRP also did not significantly reduce the number of viable bacteria of *S. aureus* after 24 h; however, it significantly reduced the number of viable bacteria for the other tested bacteria. The values of antibacterial activity before and after scrub resistance test were identical for *B. cereus* and *E. faecalis*, however, for *E. coli* a decrease (1.3) in antibacterial activity was detected after scrub resistance test. Interestingly, the antibacterial activity for *S. aureus* and for *K. variicola* increased after scrub resistance test. The increase of antibacterial activity of TRP after scrub resistance test may be related with its low aqueous solubility. Since TRP has low solubility, during the 24 h of incubation with the bacteria the amount of TRP released from the paint may be low; however, during the scrub resistance test the process of scrub may have increased the amount of TRP releasing from the paint, and thus causing a higher antibacterial effect [38].

For CLX+TRP the same tendency was verified, as this sample significantly reduced the number of CFUs/cm^2^ for *E. coli*, *B. cereus*, *E. faecalis*, and *K. variicola*. In general, CLX+TRP reduced the values of antibacterial activity after scrub resistance test for all the tested bacteria, remaining with antibacterial activity for all bacteria except *E. coli* (R = 1.5). Following scrub resistance test, CLX+TRP was not always the paint with better antibacterial performance as observed before scrub resistance test. After scrub resistance, TRP presented higher antibacterial activity against *S. aureus*, *E. coli*, and *E. faecalis*. CLX+TRP presented higher antibacterial activity against *B. cereus* and *K. variicola*.

Regarding the cytotoxicity assessment, the results of the tests by direct contact performed in HaCaT cells revealed that the developed paints presented cellular viability values over 70%, the established thresholds according to ISO 10993:5 and LDH releases under 30% [23]. Besides NRU assay, the standard ISO 10993:5, recommends MTT or XTT assay to evaluate cell viability; however, we performed the analogous assay WST-1, since it evaluates the same parameter and has less interferences and produces more accurate results [39]. In addition, we realized the LDH assay, which evaluates cell lysis. Besides giving information about the influence of the paints in the integrity of cells’ membrane that is very relevant for our study, this assay is performed in the supernatant of the cells, oppositely to the other assays, WST-1 and NRU, which are performed with the cells. This way, is possible to multiplex our assay, obtaining more information regarding the cytotoxicity of the paints and maintaining the same number of plates seeded with cells [31,40]. 

According to WST-1 assay, TRP was the paint with the lowest cellular viability in HaCaT cells after direct contact. The exposure of HaCaT cells to this paint provoked a statistically significant reduction on cellular viability. However, the results of NRU assay revealed higher values of viability, compared with WST-1. In the NRU assay, the reduction in cellular viability caused by TRP was not statistically different from the negative control. These differences in cellular viability depending on the assay (WST-1 or NRU) are probably related to the different endpoints of the assays used to evaluate viability. Although both assays use biochemical markers to assess metabolic activity of cells, they have different theoretical principles and measure different parameters. While WST-1 evaluates mitochondrial activity, NRU is related to lysosomal damage [31]. With this being, the difference in the results may be associated with the different parameters measured by each assay, suggesting that although the cells present a reduction on their mitochondrial activity after direct contact with the paints, their lysosomes are only slightly affected. 

The CLX paint also presented higher cellular viability in both assays. Weldrick et al. already proved that CLX was well tolerated by HaCaT cells after 24 h compared with other antiseptics such as Cetrimide or Benzylkonium chloride [33]. Notwithstanding, CLX presented higher values of cellular viability with WST-1 than with NRU assay; however, CLX+TRP presented similar values in both assays. Regarding LDH assay after direct contact, all the paints presented LDH release significantly higher than the negative control, as expected, though without reaching the limit of 30%. The paints CLX and CLX+TRP were also significantly different from the Un_Paint, with lower values of LDH release. CLX+TRP paint was also significantly different from TRP, displaying the lower percentage of LDH release, 13.93 ± 2.13%.

In general, the paint containing the mixture CLX+TRP was the formulation with the most promising results on the test by direct contact. This paint presented higher values of cellular viability regardless of the method used, WST-1 or NRU, and it was also the sample with the best LDH results, with values even lower than the Un_Paint. The paint TRP was the sample with the lowest cellular viability and highest LDH release, uncovering less appealing results. These outcomes were somehow unexpected, since this paint incorporates a natural substance that is usually considered safe [41]. Regarding the test on extracts on HaCaT cells, all paints presented values of cellular viability above 70% as recommended by ISO 10993:5 [23]. 

In the WST-1 assay, CLX and CLX+TRP presented a dose-dependent effect after exposure to the extracts. TRP did not reveal a gradient-concentration effect as displayed for the other paints. However, this paint presented higher standard deviations that probably affected this effect. TRP was also the sample that presented the most distinct results comparing WST-1 and NRU assays; nonetheless, this outcome may be associated with the high standard deviations obtained for this sample in the WST-1 assay. 

According to the NRU assay, the results showed a similar tendency compared to WST-1; however, more significant differences were found. In the NRU assay, all the paints, in all extracts’ concentrations, presented significant decreases in cellular viability compared with the control, though without going under the established limit of 70%. Besides, it was possible to identify a pronounced dose-dependent response according to the extracts’ concentration for all the paints.

The obtained results for LDH assay with HaCaT cells were below the established limit of 30%. CLX+TRP presented particularly interesting results, with very low levels of LDH leakage, with values varying from 3.93 ± 1.59% for the 25% extract to 8.69 ± 0.95% for the 100% extract.

The microscopic observation of the cells after direct contact exposure to the paints revealed a reduction on cellular density, namely on areas more proximate to the paint samples. This outcome was already verified in a previous study we developed with other paint samples. The smooth but unavoidable movements that the samples suffer during the assay and incubation time with the cell layer appear to affect cell adhesion and sometimes even remove some of the adhered cells on more proximate areas to the samples. This way, a lower cellular density is detected on these areas, albeit accordingly to the results of WST-1 and NRU assay, without affecting the cellular viability [29].

Regarding the test on extracts performed with A549 cells, the results obtained with WST-1 revealed that all paints, in all extract concentrations, presented high levels of cellular viability, with values over 90%. However, no dose-dependent responses were verified; rather, only very small differences between the different concentrations of extracts within the same paint were detected. 

In the NRU assay, all samples revealed acceptable values of cellular viability (above 70%). In NRU with A549, a light dose-dependent effect was also detected. 

Regarding the LDH assay on A549 cells, all the samples presented LDH leakages under the established limit of 30%. In the LDH assay with A549, as verified in the other assays performed with the same cells, no obvious dose-dependent responses were detected, with only sparse differences between extracts’ concentration within the same paint.

Comparing the two cell lines, in general, the tests on extracts realized with A549 cells revealed results somewhat different from HaCaT cells. While the assays performed in HaCaT cells verified dose-dependent responses related to the increase in extracts’ concentrations, in the assays involving A549 this effect was not so obvious, suggesting that the different cell lines have different behaviors when exposed to similar conditions. The differences in the behaviour of the two cell lines towards the same extracts’ exposure may have different causes. First, HaCaT cells are normal cells, while A549 cells are a cancerous cell line, so normal and cancerous cells present many differences, namely on the metabolism, morphology, and permeability. These factors may affect their response upon exposure to certain substances [42]. Besides, other studies have already demonstrated the differences between these cell lines when facing the same exposure [43,44].

The assay that presented the most distinct results when comparing the two cell lines was the LDH membrane integrity assay. It is important to highlight that the basal LDH releases from cells when incubated only with complete medium, and therefore, in ideal conditions, was different in A549 and HaCaT cells. In HaCaT cells, the LDH release after exposure to complete medium was around 5.28 ± 0.48%, while for A549 cell line the release was around 11.72 ± 1.76%. HaCaT cells revealed a lower residual value of LDH release that increased proportionally with the extracts’ concentration. On the other hand, A549 presented a higher basal value of LDH release that remained more constant with the increase on extracts’ concentration, suggesting the low effect of extract concentrations on LDH release from A549 cells. This may be the main factor causing such differences in LDH release values when comparing the two cell lines.

In general, the self-disinfecting paints, after extracts exposure, presented similar or higher values of cellular viability compared with the Un_Paint, and lower values of LDH release, suggesting that the immobilization of these antimicrobial substances on the original paint did not negatively influence the behavior of the cells.

Concerning the genotoxicity assessment by comet assay, for HaCaT cells, there were verified significant increases in primary DNA damage after exposure to the paints. A very marked dose-dependent effect was verified for HaCaT cells, with original extracts displaying higher values of DNA damage than the 25% extracts.

For this cell line, CLX was the sample with lower % tail intensity, presenting values even lower than the Un_Paint in both concentrations (reaching statistical differences). In the paint with mixture of the two substances, CLX+TRP, statistical differences to Un_Paint for both concentrations (25% and 100%) were also verified. While on 25% extracts a decrease was detected for the CLX+TRP compared with Un_Paint, an increase in single-strand DNA breaks was observed for the original extract (100%) in CLX+TRP. 

For the A549 cells, a dose-dependent effect was also verified. After exposure to the paints, there were increases in primary DNA damage detected, compared with the negative control, however, for some samples (TRP25% and CLX+TRP25%) without statistical differences. 

For the A549 cell line, the paint with the highest damage was CLX100%, displaying statistical difference from the Un_Paint100%. On the other hand, the paint that presented the lowest values of primary DNA damage was the mixture CLX+TRP, which presented a percentage of tail intensity even lower than the Un_Paint (without statistical significance).

Comparing the two cell lines, a similar concentration–gradient effect was observed for both. However, the response to the different paints’ exposure was different for the cell lines. While HaCaT cells presented lower primary DNA damage for CLX, A549 presented lower DNA damage for CLX+TRP. Besides, statistical differences were found when comparing the two cell lines for the samples Un_Paint100%, CLX100%, and CLX+TRP100%.

The selection of the substances CLX and TRP to be incorporated in the acrylic water-based paint, previously used in our other studies [28,45], is related to the fact that these are well-studied and well-characterized substances, with proven antimicrobial activity [35,46]. Besides, both substances are frequently used in hygiene products, disinfectants, and cosmetics since they present low toxicity when in lower concentrations [4,41]. This way, the paints developed in this work may be serve as a potential strategy to be incorporated in the antimicrobial paints’ market as they are water-based, emit less volatile organic compounds, and do not present increased toxicity compared to the acrylic paint used as control [47].

## 5. Conclusions

According to the obtained results, it is possible to conclude that a successful immobilization of the antimicrobial substances, CLX, TRP, and CLX+TRP, on an acrylic water-based paint was achieved. The paints presented antibacterial activity against most of the tested bacteria and this antibacterial effect was maintained even after drastic scrub resistance test. The paints also presented acceptable results of cytotoxicity and genotoxicity, suggesting their safe application. 

The methodologies we used to evaluate the cytotoxicity of the paints, based on ISO 10993, demonstrated good potential for evaluation of paints with self-disinfecting properties. The assays applied in this work, WST-1, NRU, and LDH, in general, presented coherent results between them and were performed without problems or interferences in the selected cell lines. Regarding the genotoxicity assessment, the antibacterial paints presented acceptable values of primary DNA damage when compared with the control paint, Un_Paint, for both cellular models, i.e., HaCaT and A549. 

This study remarkably contributes to the demonstration that paints with self-disinfecting properties may be an efficient and safe strategy to prevent surface contamination. Moreover, this study contributes to the development and implementation of methodologies to evaluate the efficacy and toxicity of antimicrobial paints, and/or other materials and coatings with antimicrobial properties, towards human cell models, as this is an important procedure in the validation of every antimicrobial product to be commercialized. Nonetheless, the in vitro toxicity assessment performed in this work still presents some limitations. In the future, it would be important to go further on the in vitro toxicity assessment using other cell lines adequate to the study and using more complex and realistic cellular models, for example 3D models, to better understand the interaction between the paints and the human cells. Regarding the antimicrobial efficacy assessment, an important future step would be to evaluate the long-term efficacy of the paints when applied in real scenarios, outside of the laboratory.

## Figures and Tables

**Table 1:** Antibacterial activity (R) values obtained for each bacterium after contact with Chloroxylenol (CLX), Terpineol (TRP), or CLX+TRP samples, before and after scrub resistance test.

		*S. aureus*	*E. coli*	*B. cereus*	*E. faecalis*	*K. variicola*
Before Scrub Resistance Test	CLX 0.15 g/L	3.4	3.5	3.4	2.5	4.0
	TRP 6.0 g/L	2.3	2.9	3.1	2.1	0.6
	CLX+TRP 3.0 g/L	4.0	2.8	4.3	3.9	6.3
After Scrub Resistance Test	CLX 0.15 g/L	2.1	1.6	3.2	2.4	3.1
	TRP 6.0 g/L	3.8	1.6	3.2	2.4	3.2
	CLX+TRP 3.0 g/L	2.8	1.5	3.3	2.2	3.9

## Data Availability

The data presented in this study are available on request from the corresponding author.

## Supplementary Materials

The following supporting information can be downloaded at: https://www.mdpi.com/article/10.3390/toxics10070343/s1, Figure S1: Microscopic images (100× magnification) of the HacaT cells with complete medium used as negative control (C−) or after 24 h of incubation in direct contact with the samples transparent polymeric film (W), Cooper (Cu^2+^), Un_Paint, CLX, TRP or CLX+TRP.
