# Supplementary material for: Development and In Vitro Validation of Antibacterial Paints Containing Chloroxylenol and Terpineol
Source: Toxics. 2022 Jun 21;10(7):343. doi: 10.3390/toxics10070343 (PMC9324826; doi:10.3390/toxics10070343)
Supplement: Supplementary file 1 [file toxics-10-00343-s001.zip › toxics-1744065-supplementary.pdf]

toxics

Supplementary Materials: Development and In Vitro Validation of Antibacterial Paints Containing Chloroxylenol and Terpineol

Micaela Machado Querido, Ivo Paulo, Sriram Hariharakrishnan, Daniel Rocha, Nuno Barbosa, Diogo Gonçalves, Rui Galhano dos Santos, João Moura Bordado, João Paulo Teixeira and Cristiana Costa Pereira

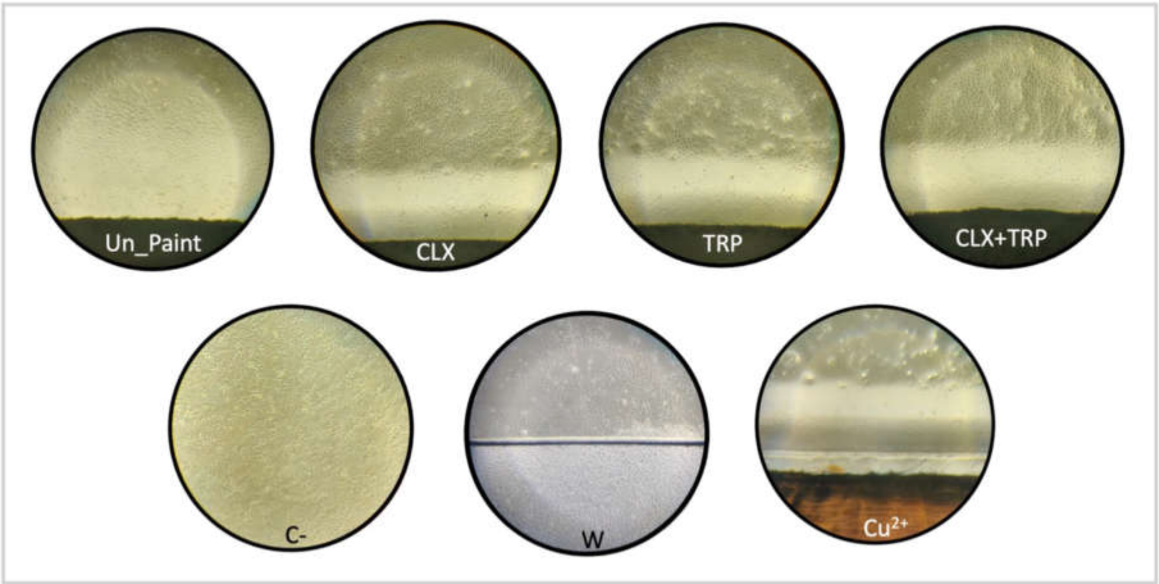

Figure S1. Microscopic images (100× magnification) of the HacaT cells with complete medium used as. negative control (C-) or after 24 h of incubation in direct contact with the samples transparent polymeric film (W), Cooper (Cu^{2+}), Un_Paint, CLX, TRP or CLX+TRP.
